# Histamine food poisoning: a sudden, large outbreak linked to fresh yellowfin tuna from Reunion Island, France, April 2017

**DOI:** 10.2807/1560-7917.ES.2019.24.22.1800405

**Published:** 2019-05-30

**Authors:** Guillaume Velut, François Delon, Jean Paul Mérigaud, Christelle Tong, Guillaume Duflos, François Boissan, Stéphanie Watier-Grillot, Mickaël Boni, Clement Derkenne, Aissata Dia, Gaëtan Texier, Philippe Vest, Jean Baptiste Meynard, Pierre Edouard Fournier, Aurélie Chesnay, Vincent Pommier de Santi

**Affiliations:** 1 French Military Health Service, French Armed Forces Centre for Epidemiology and Public Health (CESPA), Marseille, France; 2 French Military Health Service, 28th Veterinary Group, Paris, France; 3 Laboratory for Food Safety, Department of Seafood and Aquaculture, ANSES (French Agency for Food, Environmental and Occupational Health & Safety), Boulogne Sur Mer, France; 4 French Military Health Service, 2^nd^ Armed Forces Medical Centre, Versailles, France; 5 French Ministry of Defense’s Joint Logistics and Supply Agency (Économat des armées), Pantin, France; 6 Paris Fire Brigade, Paris, France; 7 UMR VITROME, Aix-Marseille University, IRD, SSA, AP-HM, IHU-Méditerranée Infection, Marseille, France; 8 French Military Health Service, Biomedical Laboratory, Percy Military Teaching Hospital, Clamart, France; 9 Laboratory of the French Armed Forces Commissariat, Angers, France

**Keywords:** histamine, outbreak, poisoning, military personnel, tuna, fish, France

## Abstract

On 20 April 2017, an outbreak of histamine food poisoning occurred in a French military unit located near Paris. A total of 40 cases were identified (attack rate: 16.6%). We conducted a case–control study on 31 cases and 63 controls. Multivariate analysis pointed to cooked yellowfin tuna fillet as the very likely source of food poisoning (odds ratio = 156.8; 95% confidence interval: 18.4–1,338.4). The fresh yellowfin tuna was from Reunion Island and was supplied vacuum-sealed and packed with ice at the principal food market of Paris. No cold chain issues could be established in the upstream and downstream supply chains. Histamine concentration was found to be 1,720 mg/kg in leftover raw tuna, and 3,720 mg/kg in control cooked tuna, well above the threshold limit values defined by European regulations (200 mg/kg). The presence of *Klebsiella variicola* and *Pantoea agglomerans,* microorganisms of the Enterobacterales order that have been reported to produce histamine, was confirmed in the leftover raw tuna. This type of food poisoning is rarely recognised and confirmed. We describe the outbreak to highlight the specific key points of this type of investigation.

## Introduction

Histamine food poisoning (HFP) is an allergy-like reaction caused by consumption of fish or fermented foods containing a high concentration of histamine [1]. The clinical picture is usually characterised by rapid onset (within 1 hour) of symptoms such as flushing, cutaneous rash or headache; mild severity; short duration and self-limitation [2]. Scombroid fish (e.g. tuna, mackerel) and some non-scombroid fish (e.g. mahi-mahi, sardines, pilchards, herring) are commonly implicated in HFP. Their muscles contain a high level of free amino acid histidine, which is preserved as a substrate for bacterial histidine decarboxylase [3]. Through this enzyme, certain bacteria can form histamine from histidine. These bacteria can come from normal fish flora (in particular, fish gut Enterobacterales), from the marine environment and from secondary contamination of the food (improper handling, cross-contamination from catering equipment and facilities, and/or raw foods) [4]. Other biogenic amines might be produced during bacterial growth and potentiate the histamine effect by inhibiting intestinal histamine metabolising enzymes [5]. The production of biogenic amines depends mainly on time-temperature abuse (deviation from the optimal storage temperature for a time period), but also on fish product pH, salinity, oxygen availability and competition with other spoilage microorganisms [2]. Histamine production is most commonly due to inadequate refrigeration of the fish, and can occur at any stage of the food chain [6]. Once formed, histamine—and to some extent other biogenic amines—are not destroyed by cooking, smoking or freezing [7].

The proportion of HFP among food poisoning outbreaks can vary according to the dietary habits of countries. According to the European Food Safety Authority (EFSA) and European Centre for Disease Prevention and Control (ECDC), 599 HFP outbreaks were notified in the European Union (EU) during the period 2010–17. HFP reached its highest incidence in 2017, with 117 HFP outbreaks involving 572 cases, mainly reported by France and Spain [8,9]. This increased incidence was assessed by EFSA and linked to four grouped and connected multistate events [9].

According to national surveillance in France, HFP accounted for 3.6% (263/7,346) of food-borne outbreaks reported between 2010–16 [10]. Out of these HFP outbreaks, only 23.6% (62/263) were confirmed in the laboratory, which underlines the difficulties of investigating such events.

This type of food-borne outbreak is rarely recognised, is usually limited and seems to be becoming more frequent in the EU. We report a large HFP outbreak to highlight the key points of our investigation and to discuss the improvement of prevention measures.

### The event

On 20 April 2017, the medical centre of a French military unit located near Paris alerted the French Armed Forces Centre for Epidemiology and Public Health that 23 military and civilian staff members had shown signs of poisoning within minutes of having lunch at the same catering facility. Given the unexpected nature of the situation, a pre-hospital emergency response team involving 23 vehicles and 58 rescue members was deployed. The first reported symptoms were headache and cutaneous rash. Four patients had sinus tachycardia and two patients had hypotension, one of which required intravenous fluid therapy. All patients reported eating tuna at lunch. Nineteen patients were transferred to local emergency departments for observation and were discharged within 3 hours. This event occurred in the context of a heightened risk of terrorist attacks in Paris. In order to exclude an intentional or malicious act, sample meals were requisitioned by the national gendarmerie and local judicial officials requested the results of the investigation. However, HFP was quickly suspected due to the allergy-like symptoms of the patients, the rapid onset after a meal and the consumption of fresh yellowfin tuna.

## Methods

The outbreak investigation was carried out by a multidisciplinary team including clinicians, biologists, epidemiologists and veterinarians who are in charge of food safety in the French Armed Forces.

### Epidemiological investigation

Due to the wide diversity of symptoms caused by histamine poisoning, a sensitive case definition was considered the most appropriate. A case was defined as a service member who ate lunch at the military catering facility on 20 April 2017 and who presented, within 3 hours after eating, with at least one of the following symptoms: headache, hot flushes, rash, nausea, vomiting, palpitations, diarrhoea, abdominal pain, itching or a burning taste sensation.

Active case detection was performed among staff (including kitchen staff) who ate lunch at the military unit’s catering facility. The case–control survey was organised among staff who ate lunch at the military catering facility on the following Monday (24 April 2017). All staff that met the case definition, as well as two controls per case, completed a short self-administered questionnaire. Controls were selected among staff members who ate lunch at the military catering facility on 20 April 2017, had none of the aforementioned symptoms and were available for an interview.

Univariable analyses were performed by Pearson’s chi-squared test and Fisher’s exact test when required. Variables with a p value < 0.1 were selected for multivariable logistic regression modelling. A manual backward selection procedure was used with a cut-off of p < 0.05, using R software, version 3.3.3 (R Foundation for Statistical computing, Vienna, Austria).

### Laboratory investigation

#### Blood samples

Blood samples from six hospitalised patients were tested for histamine and tryptase plasmatic concentration, respectively, using the radioimmunoassay method and ImmunoCAP Tryptase (Thermo Fisher Scientific Inc., Waltham, United States (US)).

#### Food samples

Food samples from all the dishes served at the lunch service were taken for bacteria and toxin analyses. There were two types of fish samples: cooked yellowfin tuna fillet (SO1) and a mix of raw offcuts (more than 25 pieces) from the different tuna loins composing the supply (SO2). The samples were sent to the Laboratory of the French Armed Forces Commissariat in order to determine the histamine concentration of the tuna (VERATOX Histamine Kit, Neogen Corp., Lansing, US) and to perform bacterial isolation (Petrifilm). As a second step, the samples were sent to the Laboratory for Food Safety, Department of Seafood and Aquaculture, to look for other amines using the internal method LSA-INS-0017, adapted from method previously described [11]. In order to evaluate the freshness of the fish, the quality index was calculated using the formula given by Mietz and Karmas et al.: (histamine + putrescine + cadaverine) / (1 + spermidine + spermine) [12]. Enterobacterales identification was performed by the Méditerranée Infection Hospital-University Institute using matrix-assisted laser-desorption/ionisation time-of-flight (MALDI-TOF) mass spectrometry for MS protein analysis and 16S rRNA sequencing using fD1-rP2 primers, as previously described [13,14].

### Trace-back investigation and control measures

The veterinary service collected information about the meals served at the military catering facility and requested the immediate withdrawal of the remaining batch of tuna. After checking the tuna delivery and labelling slips, the Departmental Directorate for the Protection of Populations was informed of the situation and of the batch number under investigation. The caterer was identified, and inspection of the catering process and review of food hygiene procedures were carried out on site.

Upstream supply chain traceability information was requested from the seller at Paris’ principal food market, whose facilities were also checked on the next delivery.

### Ethical statement

All case–control study participants received information about the investigation and the disease, and participated voluntarily. According to French regulation, as this was a severe food-borne outbreak with immediate public health threat, no ethical approval was required.

## Results

### Epidemiological investigation

**Figure 1:**
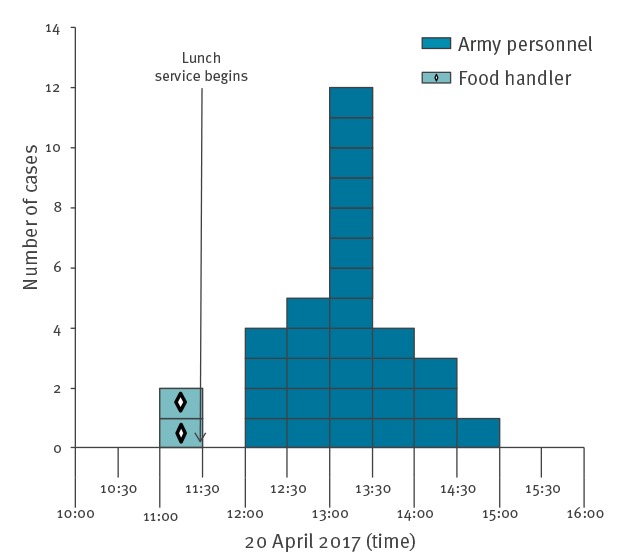
Cases in histamine food poisoning outbreak by onset of symptoms, France, 2017 (n = 31 cases)

**Figure 2:**
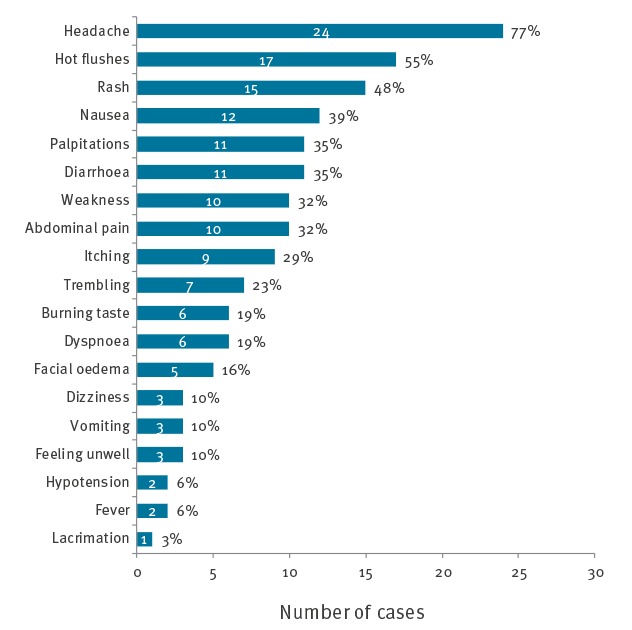
Symptoms presented by cases in histamine food poisoning outbreak, France, 2017 (n = 31)

A total of 40 food poisoning cases were identified among the 241 people who had eaten at the military catering facility on 20 April 2017, leading to an attack rate of 16.6% of those who had eaten lunch and 49.4% of those who had eaten tuna (39/79). Of these 40 cases, 23 were initially identified and 17 were found by active case detection. Ninety-four individuals were included in the case–control study, 31 cases and 63 controls. The median age of cases was 38 years (18–58 years) and 68% (21/31) were men. The first cases occurred shortly after 11:00, in two food handlers who had eaten before lunch. The majority of cases had symptom onset between 13:00 and 13:30 (Figure 1). The median incubation period was 60 minutes (range: 0–135 minutes). The most common symptoms were headache (77%), hot flushes (55%), rash (48%), nausea (39%), palpitations (35%) and diarrhoea (35%) (Figure 2).

Cases may have had several of the listed symptoms.

Among cases, 97% (30/31) had consumed tuna, whereas only 19% (12/63) of controls had. In univariable analysis, yellowfin tuna fillet (odds ratio (OR) = 127.5; 95% confidence interval (CI): 15.8–1029.9, p < 0.001) and cod fish fritters (OR = 4.3; 95% CI: 1.6–11.5, p = 0.004) were the only consumed foods suspected of being the source of contamination. However, the consumption of the cod was associated with that of the tuna (OR = 4.8; 95% CI: 1.9–12.18, p < 0.001). Multivariable analysis confirmed that only tuna consumption was a risk factor for food poisoning (adjusted OR = 156.8; 95% CI: 18.4–1338.4, p < 0.001) (Table 1).

**Table 1:** Histamine food poisoning outbreak, case–control study, univariable and multivariable logistic regression analysis, France, 2017 (n = 94 cases and controls)

Food items	Cases (n = 31)	Controls (n = 63)	OR (95% CI)	p value	aOR (95% CI)	p value
	n	n				
Cod fish fritters	24	25	4.3 (1.6–11.5)	0.004	NA	NA
Pepper salami	1	12	0.1 (0.003–0.9)	0.03	0.08 (0.007–0.9)	0.04
Melon	1	14	0.1 (0.002–0.8)	0.02	NA	NA
Mushroom salad	5	10	0.9 (0.3–3.0)	0.89	NA	NA
Radishes	1	4	0.4 (0.008–4.5)	0.65	NA	NA
Grated carrots	7	12	1.1 (0.4–3.2)	0.84	NA	NA
Turkey colombo	2	41	0.03 (0.003 -0.1)	< 0.001	NA	NA
Yellowfin tuna fillet	30	12	127.5 (15.8–1029.9)	< 0.001	156.8 (18.4–1338.4)	< 0.001
Madras rice	20	47	0.5 (0.2–1.3)	0.16	NA	NA
Grilled eggplant	13	25	0.9 (0.4–2.2)	0.86	NA	NA
Cheese	11	24	0.8 (0.3–1.9)	0.54	NA	NA
Coconut macaroon	12	19	1.1 (0.5–2.8)	0.79	NA	NA

aOR: adjusted odds ratio; CI: confidence interval; OR: odds ratio; NA: not applicable.

### Laboratory investigation

#### Human samples

It was possible to obtain blood samples for six hospitalised patients, 4 hours after the onset of symptoms. Of these patients, two had abnormal plasmatic histamine concentration of 49.3 nmol/L and 25 nmol/L (norm : < 10 nmol/L). Plasmatic tryptase concentration was normal or near normal for all the patients (norm: < 1.5).

#### Food samples

Histamine concentration was found to be 1,720 mg/kg in the raw offcuts of yellowfin tuna (SO2) and 3,720 mg/kg in the cooked tuna fillet (SO1), which is well above the threshold limit values set by European regulations (200 mg/kg) [15]. Further analyses on the tuna samples were performed separately to assess the variation of histamine concentration, which ranged from 3,080–7,200 mg/kg (median: 3,440 mg/kg) for raw offcuts of tuna (five samples) and 840–4,140 mg/kg for cooked tuna (two samples). Other biogenic amine concentrations were also elevated (Table 2).

**Table 2:** Bacteriological and bioamine results for raw and cooked fresh yellowfin tuna, histamine food poisoning outbreak, France, 2017

Bacteria and bioamines	Raw tuna (SO2)	Cooked tuna (SO1)
*Escherichia coli*	< 10 CFU/g	< 10 CFU/g
Enterobacteria (30 °C)	5,500 CFU/g	< 10 CFU/g
Enterobacteria (37 °C)	2,400 CFU/g	< 10 CFU/g
Isolated bacteria	*Klebsiella variicola* *Pantoea agglomerans*	NA
Histamine	1,720 mg/kg	3,720 mg/kg
Putrescine	87 mg/kg	54 mg/kg
Cadaverine	38 mg/kg	10 mg/kg
Tyramine	14 mg/kg	15 mg/kg
Spermidine	13 mg/kg	7 mg/kg
Spermine	37 mg/kg	17 mg/kg
Quality index	51	76

NA: not applicable.

The quality index was 51 for the raw tuna and 76 for the cooked tuna, well above the limit of fish acceptability (limit = 10) retained by Mietz and Karmas [12]. Bacterial contamination was found on leftover raw tuna with an Enterobacterales count of 5,500 CFU/g, but not in the cooked tuna. The presence of *Klebsiella variicola* and *Pantoea agglomerans* was confirmed by MALDI-TOF and PCR techniques.

### Trace-back investigation and control measures

As soon as HFP was suspected, all the remaining uncooked tuna was withdrawn from the military catering facility. According to the Departmental Directorate for the Protection of Populations, tuna from this same batch had not been delivered to other purchasers. This tuna, which came from Reunion Island, arrived by air on 17 April 2017 at 19:00 at Roissy-Charles de Gaulle airport and was delivered on the morning of 19 April 2017 to the food market in Paris. One screening test was performed by the local supplier in Reunion before transport to France, with histamine concentration observed under 200mg/kg, and was not repeated afterwards. The fresh tuna was vacuum-sealed and packed with ice, with five loins per polystyrene package (ca 15 kg per package). Recorded temperature values could be traced from the fishing boat to the self-catering military facility; they complied with the regulatory requirements (temperature between 0 °C and + 2 °C for raw fishery products) [16]. Assessment of the catering process and facilities did not highlight any deviation from food storage and hygiene practices. No abnormal odours or changes in the appearance of the tuna were observed by the cooks. The fish was first quickly fried and then baked in the oven.

## Discussion

Several aspects of this investigation were unusual. First, the large size and sudden occurrence of the outbreak, which explain the large-scale emergency service response. Then, suspicion of poisoning in a military population initially raised the issue of a potential intentional attack. However, it was possible to dismiss this hypothesis in light of the rapid and favourable resolution of symptoms; the consumption of tuna, which was statistically strongly associated with histamine poisoning; and confirmation of histamine in the food samples. As a limitation, nine cases could not be included in the case–control study. However, as no cases were hospitalised for more than 3 hours, it was unlikely that their absence during the case–control study was related to their clinical presentation. All of these cases had eaten tuna, which would have strengthened the association with tuna, if they had been included.

HFP can sometimes lead to severe cases or death [17-19], even among healthy patients [20,21]. Therefore, suspected or confirmed HFP should be considered an emergency and confirming the origin of HFP should be made an urgent priority, in order to protect other consumers.

Tuna species, especially yellowfin tuna, are frequently implicated in HFP [22-25]. In this outbreak, the initial onset of symptoms in two food handlers could have alerted staff early on; however, their symptoms (pruritus) were, to them, unexpected for a food-borne outbreak and therefore were not immediately associated with the tuna. To limit the consequences of food poisoning or intentional food contamination, procedures should include ceasing the distribution of suspicious dishes as soon as allergic or other unusual symptoms (respiratory, neurological, etc.) appear among consumers and immediately alerting the food safety authorities.

Two of six patients in this HFP outbreak had high levels of histamine and normal or near-normal plasma tryptase levels. These results were consistent with HFP, but were not sufficient to confirm it, mainly due to the delay before sampling. Indeed, although plasma histamine levels increase soon after the ingestion of contaminated fish, it has a short half-life (2 minutes) and blood samples need to be taken within 4 hours [26]. In addition, the plasma histamine level can increase in stressful conditions, leading to excess errors [27]. Normal plasma tryptase levels exclude anaphylaxis, but blood samples must be taken 1–6 hours after the exposure [28,29]. Thus, these examinations are useful only if sampling can be carried out 1–4 hours after the suspected poisoning. In addition, the results must be compared with the basal level, measured at least 24 hours after symptoms.

The case–control study strongly pointed to the cooked yellowfin tuna fillet that was eaten the day of the HFP outbreak. Therefore, no further analysis was performed for the cod fritters or dishes with an OR <1. In food-borne outbreaks where HFP is suspected, conducting a case–control study can help to confirm the link with the food product, especially if several of the served dishes carry a risk for HFP (e.g. other seafood products, fermented foods and cheeses) [30].

It is essential to confirm HFP by verifying that there is a very high level of histamine concentration in the incriminated foodstuff. In our study, the histamine concentration in the raw and cooked tuna was clearly above the European regulatory values (maximum of 200 mg/kg in raw fish). However, concentration may vary significantly in the flesh of one tuna or between different fish in one batch [23]. This could explain why not all the people who ate the tuna became sick and why the analysis performed by the local supplier in Reunion was normal. Therefore, to confirm histamine presence in suspected food, several samples must be analysed.

The other biogenic amine levels in the tuna were also elevated and might have potentiated the effects of histamine. There is no regulatory limit for the level of other biogenic amines, limiting the interpretation of their contribution to HFP. However, some authors have proposed using a composite index to evaluate the freshness of fish; in our samples, the quality index was very elevated, suggesting an advanced state of spoilage [11,31]. More studies are needed to evaluate the relevance of detection and cut-off levels of other biogenic amines to prevent HFP.

The two bacteria isolated in the raw tuna—*Klebsiella variicola* and *Pantoea agglomerans—*have been described in another HFP outbreak as being able to produce histamine [32]. Some specific strains (4G2–1 and LC1–8) of *Pantoea agglomerans* have been proven to be prolific histamine formers [33,34]. We suspect that the most likely source of histamine production in this HFP outbreak was the presence of these bacteria. These two species are known to be facultative anaerobes able to grow in reduced oxygen environments. *Pantoea agglomerans* has been found in vacuum or modified-atmosphere (60% CO_2_ and 40% N_2_) packed herring under 2 ± 2◦C storage conditions [35]. Vacuum packaging is used to increase the shelf life of fish products; it is considered, as is modified-atmosphere packaging, to be generally more effective in inhibiting or delaying the production of histamine than air packaging [36]. Nevertheless, these preservation methods can allow for the growth of anaerobe bacteria that produce histamine, which may call into question the effectiveness of these measures. Descriptions of regional and global trends in HFP-isolated bacteria and the packaging techniques involved should be considered in further studies.

In this HFP outbreak, another factor that might have enabled histamine production is that these bacteria grow best at moderate temperatures. Some strains of *Pantoea agglomerans* are able to grow at 4 °C [37], and some *Pantoea* species have shown a cold growth of 6.57 (log day 10−log day 0) at 6 °C [38]. Thus, even a small temperature shift could have contributed to this HFP outbreak.

Even if fraud was hypothetically possible, no time-temperature abuse in the cold chain was identified in our study. Our assumption is that the contamination may have occurred early in the fishing phase (time spent in the water after death) or during the initial preparation of the fish (cutting, evisceration).

Finally, we underline that only one histamine-screening test was performed throughout the fish supply chain. This single test, administered by the supplier, did not identify histamine contamination. In France, histamine analyses are performed by authorities only on imported products from non-EU countries, and Reunion is part of the EU. The cost of screening tests could limit repeated analysis by suppliers and sellers. As recommended by the Food and Agriculture Organization of the United Nations and by the World Health Organization [3], standardised procedures and more widespread use of histamine rapid testing would improve the screening of fish products, especially for fish coming from warm seas and/or when there are long transport delays.

### Conclusions

No severe cases occurred during this HFP outbreak, but the military population is usually healthier than the general population. This type of food poisoning could have more serious consequences for other populations of fish consumers. European fresh tuna imports increased by 5% per year on average between 2011–15 [39]. If this trend continues, the risk of HFP could increase in the coming years, as observed in 2017. In this context, our HFP investigation underlines that: (i) a sudden outbreak of allergy-like symptoms must immediately prompt suspicion of HFP and lead to a rapid withdrawal of the suspected food, (ii) human samples are not useful unless taken within 4 hours after symptom onset and must be retested after 24 hours, (iii) epidemiological investigation and biogenic amine detection in the incriminated food are crucial to confirming the link between the outbreak and the source of the poisoning, (iv) the identification of histamine-forming bacteria can underpin the causal link and may help to improve prevention measures and surveillance information and (v) cold-chain and handling practices must be monitored and traced from fish capture to the consumer’s plate. Furthermore, this type of investigation cannot be successful without a strong and multidisciplinary collaboration involving physicians, biologists, epidemiologists and food safety specialists.
